# Data and cooperation required for Venezuela’s refugee crisis during COVID-19

**DOI:** 10.1186/s12992-020-00635-7

**Published:** 2020-10-22

**Authors:** Claire J. Standley, Eric Chu, Emrose Kathawala, Deisy Ventura, Erin M. Sorrell

**Affiliations:** 1 Center for Global Health Science and Security, Georgetown University, Washington DC, USA; 2 Department of Microbiology and Immunology, Georgetown University, Washington DC, USA; 3 Walsh School of Foreign Service, Georgetown University, Washington DC, USA; 4 Public Health School, University of Sao Paulo, São Paulo, Brazil

**Keywords:** Venezuela, Migrant crisis, COVID-19, Cross border infectious diseases, Health systems

## Abstract

The deteriorating political and economic situation in Venezuela has ramifications far beyond the Latin American country’s borders as almost five million Venezuelans fled and migrated into countries in the region due to the crisis at home. The scarcity of health services, the lack of information sharing, and the absence of reliable data in Venezuela create challenges for confronting developing health emergencies and disease outbreaks. The need for accurate data is especially dire given the current COVID-19 pandemic and evolving movement of refugees. While countries and international organizations came together to form a coordinated response to Venezuela’s political and humanitarian crisis, this geopolitical progress is threatened by the rapid spread of COVID-19, and the instinct for countries to focus inwards on domestic response priorities, rather than engage in regional cooperation. It is critical that the international community set aside geopolitical differences and cooperate to seek an accurate picture of the conditions on the ground to improve the welfare of Venezuelan migrants and to provide a more robust response to the current pandemic.

## Background

### Venezuela’s political and humanitarian crisis

On January 23, 2019, Venezuela’s opposition leader Juan Guaidó announced that the presidency of the country was “vacant” and declared himself acting president. The then 36- year old, was a member of the country’s national assembly and had been elected to lead the legislature just 18 days prior. By law, as the leader of the national assembly, Guaidó had the right to fill the presidential vacancy. To date, almost 60 governments around the world have recognized him as the legitimate president of Venezuela, including the United States and most European and Latin American countries. However, Nicolás Maduro has remained in political and military control of the country and is recognized as president by countries such as Russia, China, Iran, Syria, Cuba, and Turkey. Despite the evolving situation and long historical roots, the political and humanitarian crisis can be traced back to 6 years before Guaidó’s declaration and has resulted in extended health, economic, and geopolitical ramifications.

In April 2013, Nicolás Maduro was elected president with a 1.6% margin after his mentor and predecessor, Hugo Chávez, died in office. Tanking oil prices in 2014 hit the Venezuelan economy hard after years of mismanagement, corruption, and international sanctions. Living standards fell as Venezuelans struggled with inflation, budget deficits, food and medicine shortages, and deteriorating living conditions. Political and personal freedoms likewise suffered, as protests became illegal and press were unable to report freely. Routine human rights abuses such as torture, beatings, and arbitrary detentions were common [1, 2]. Without heeding to calls for reforms, Maduro focused instead on consolidating political power. In the 2018 presidential election, the government barred many opposition candidates from running, which resulted in Maduro’s highly controversial and disputed re-election. Venezuela’s opposition rejected the election results, eventually leading to Guaidó’s ascension and the current political crisis. Maduro has rewarded allies, including the military, with income from state industries and other illicit activities. In 2019 alone the economy contracted by 35% and inflation rose to an astounding 9585%. The International Monetary Fund (IMF) forecasts that hyperinflation will continue in 2020 and an economic contraction of some 15% could occur [3]. Venezuela’s poverty rate rose to levels unmatched across Latin America. The 2019–20 National Survey of Living Conditions (ENCOVI), conducted by researchers at Andres Bello Catholic University and published in July 2020, indicates 64.8% of households experienced “multidimensional poverty” in 2019, taking into account income and access to education and public services [4]. This number jumps to 96% when looking only at income levels. Venezuela’s main source of government revenue, crude exports, fell to their lowest levels in 75 years in 2019.

As the political crisis continues, a humanitarian crisis has already unfolded. According to a report by the United Nations High Commission for Human Rights (UNHCR), “[s] tate authorities’ ineffective measures or inaction to address the acute deterioration of health care facilities and equipment, the unavailability of medicines, in particular for patients with chronic diseases, and the outbreak of diseases that had been eradicated, led to violations of the right to an adequate standard of health of a large number of people throughout the country [2].” In response to the health emergency, multiple countries, the UN, and eventually even the Vatican offered humanitarian aid to Venezuela. However, Maduro’s government refused to acknowledge the existence of a humanitarian crisis and accept international assistance, responding in violence in February 2019 when Maduro government forces killed seven people and prevented aid from entering the country from Colombia. The regime later only accepted limited assistance from the Red Cross and the World Health Organization (WHO).

The health crisis compounded the severity of the situation. The Maduro regime refused to release information required to analyze the consequences of the health crisis, including weekly epidemiology bulletins [5]. The Venezuelan Ministry of Health did not publish official reports or release data from 2016 to 2019, making it difficult to understand and address the health issues in the country. The collapse of the Venezuelan health care system is drastic, as 14% of intensive care units are unable to operate and 79% of facilities analyzed have no water [5]. In late March, the Venezuela Government released an inventory of hospital beds, reporting 23,762 [6]; a severe overestimation as most healthcare centers are functioning at limited or zero capacity due to inadequate infrastructure, personnel, medicines and consumable [7]. Numerous publications, including the National Survey of Venezuelan Hospitals and the Venezuelan Defence for Epidemiology Network, report an estimated 720 critical care beds for the country [8, 9]. Difficulty to acquire medicines, equipment, and supplies continues to impede the ability of Venezuela’s health system to provide care. The Venezuelan Medical Federation (Federación Médica Venezolana) reports that at least 30,000 medical professionals have left the country over the last decade, contributing to a shortage of specialists [10]. All aspects of public health have been impacted by the collapse of the health system; interrupted campaigns and low coverage led to the emergence of vaccine-preventable diseases like diphtheria and measles while lags in testing and treatment caused an increase in HIV cases with the highest prevalence in the country’s second-largest indigenous populations, the Warao [11–14] . By 2018, 500,000 cases of malaria were reported in the country, even though Venezuela had previously eradicated the disease in 1961 [15].

## The scale of the migrant crisis

**Fig. 1:**
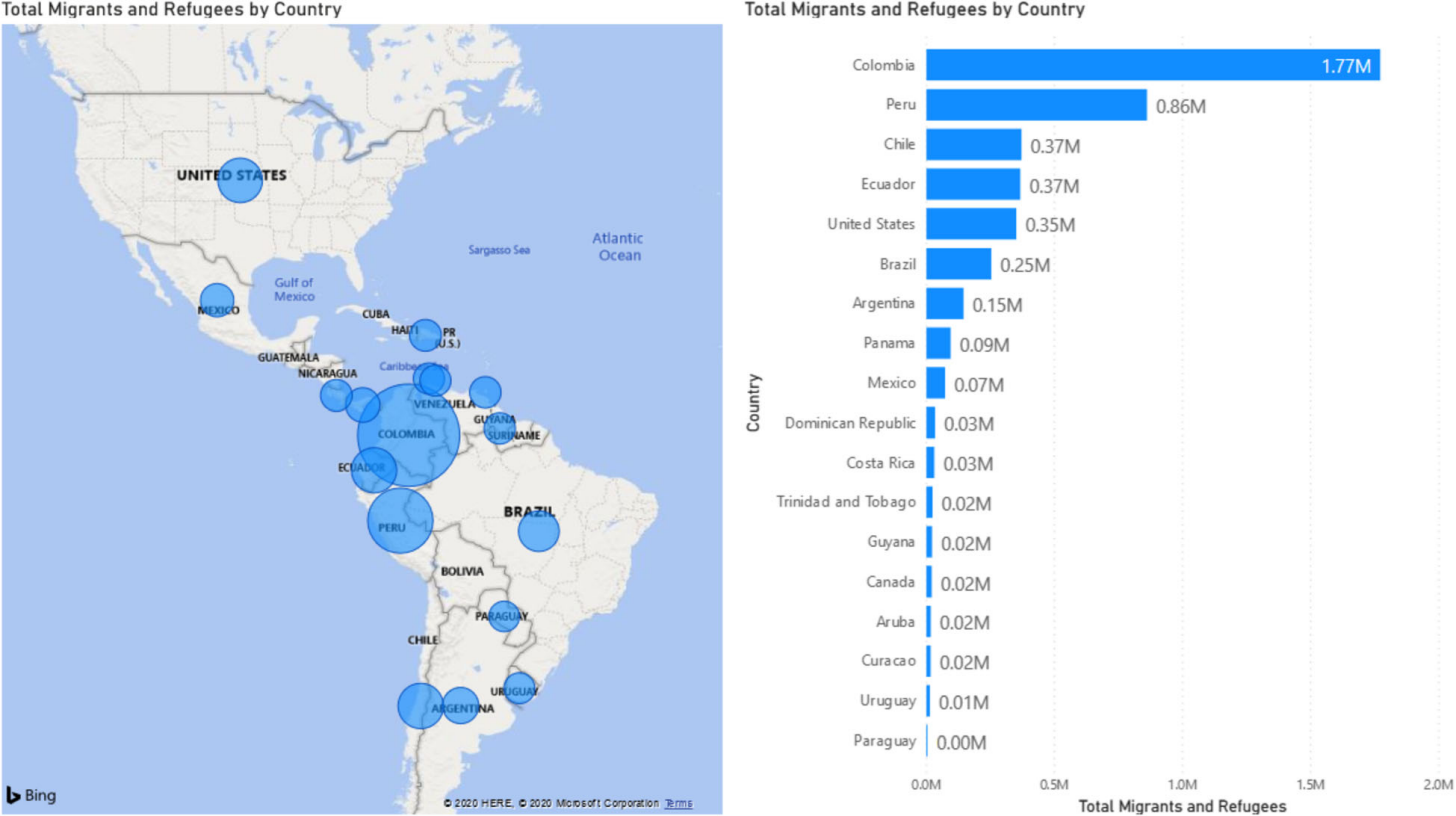
Total migrants and refugees from Venezuela in neighboring countries

Most of the people who have fled Venezuela have remained within Latin America. As of December 2019, 1.63 million Venezuelan migrants reside in Colombia, 864,000 in Peru, 385,00 in Ecuador, 371,000 in Chile, 224,000 in Brazil, 145,000 in Argentina, and at least 10,000 in other countries (Fig. 1) [16]. The influx of migrants from Venezuela into neighboring Latin American countries had been better received than previously witnessed with the influx of Syrian refugees into Europe.

### Regional health impacts from the migrant crisis

The deteriorating Venezuelan health care system and its negative repercussions for population health have become a regional issue as Venezuelans move to neighboring countries. Increased maternal mortality rates, the reemergence of infectious diseases that had previously been controlled or even eliminated, and the return of vaccine-preventable diseases are all of concern not only for migrants on the migratory path but for the healthcare systems of host countries as well [12, 17]. Neighboring countries, specifically Colombia and Brazil, have become important sources of accurate information and health data of the Venezuelans in the absence of reporting from within Venezuela. As an indicator of the added health stress neighboring countries have taken on, the number of Venezuelans seeking medical care at Colombia’s North Santander border area rose from 182 in 2015 to 5094 in 2018. More than 8000 pregnant Venezuelan women were expected to give birth in Colombia [18]. These numbers provide a partial and staggering picture of the inadequate health conditions in Venezuela.

Overcrowding, poor sanitation, and lack of access to timely and appropriate medical services have increased migrant vulnerabilities to infectious diseases and exacerbated transmission and spread. For example, Venezuela’s malaria outbreak may have crossed international borders. According to a PAHO/WHO epidemiological report, there has been an increase in malaria cases across the Americas, with Brazil, Ecuador, Mexico, Nicaragua, and Venezuela all reporting increases in 2017 [19]. Of the imported malaria cases reported in 2016, 78% of the cases in Brazil and 81% of the cases in Colombia are suspected to have originated in Venezuela, with cases in Guyana also associated with the malaria outbreak in Venezuela [20]. Across these countries, the high influx of Venezuelan migrants needing treatment has led to medicine shortages and reduced both resident and migrant access to treatment [15]. It is important to note that while this is not an indication that the migrants were carrying diseases across borders, poorly managed migration has the potential to result in disease outbreaks.

The breakdown in Venezuela’s health system has also led to an increased incidence of vaccine-preventable diseases among migrant Venezuelans. Brazil, Colombia, and Ecuador all reported cases of measles with links to Venezuelan nationals [20]. The measles outbreak in the Brazilian state of Roraima, which reported 84 cases in 2018 with 69% occurring in Venezuelan migrants, spread to the states Amazonas, Rio de Janeiro, Rio Grande de Sol, Rondônia, São Paulo, and Pará, each with cases having identical lineages to the cases reported in Venezuela [21]. Furthermore, Colombia reported two cases of diphtheria from unvaccinated Venezuelan children, and in 2017 Brazil reported a case of diphtheria in a Venezuelan migrant [20]. In 2018, due to the accelerated flow of Venezuelans and reports of serious violations of their rights, the Federal Government of Brazil created “Operation Welcome”, which is a humanitarian task force supported by UN agencies and dozens of civil society entities and coordinated by the Brazilian Army [22]. The cities of Boa Vista and Pacaraima of the State of Roraima receive the largest number of Venezuelans in occupations organized by the migrants themselves or shelters managed by Operation Welcome [23]. In these two cities, the state government decreed a public health emergency in February 2019, but despite the transfers made to mitigate the abrupt increase in demand, the infrastructure and the available material and human resources were and still are deficient [24].

To further compound the crisis, the novel coronavirus (COVID-19) pandemic is putting additional strain on health systems worldwide and certainly in Venezuela and its neighbors. Latin America comprises less than 10% of the global population yet accounts for nearly one-third of reported COVID-19 deaths. The fallout could push an additional 16 million residents of the region into extreme poverty, according to a report from the United Nations [25]. According to a survey that interviewed 390 Venezuelans, the majority living in Colombia, Chile, Peru and Ecuador, 9 out of 10 Venezuelans lost income due to the pandemic; 2 out of 3 need some help to ensure food; 1 in 4 have no money for purchases and depend solely on donations; also 1 in 4 is on the street, causing a 20% drop in remittances from abroad to Venezuela [26].

Venezuelans in Brazil suffer together with the entire population due to the disastrous response to the pandemic, marked by denialism and political conflict [27]. On August 16, 2020, Brazil documented 3.2 million cases and 106,000 deaths [28]. It should be noted that there are no national data on cases and deaths of migrants. In the State of Roraima, on August 14, 2020, 5.7% of COVID-19 deaths were Venezuelans [29]. Poor living conditions, difficulty accessing the health service, the language barrier, and the lack of employment are some of the many factors that place refugees and other migrants in COVID-19 risk groups in Brazil [30]. With a lack of documents and a closure of public offices, migrants are unable to obtain emergency aid the federal government created during the pandemic [31]. However, field hospitals, like the one created by the Operation Welcome in Boa Vista, are in service specifically for Venezuelan migrants [32].

Public health experts warn that COVID-19 transmission rates and mortality will be higher in Venezuela compared to other countries in the Americas. Even with the government-imposed quarantine, food and water shortages force people to leave their homes. The government continues to threaten physicians and health care workers with retaliation if they speak out publicly about COVID-19 cases [33]. There are valid concerns that state-imposed quarantines and lock-downs will also stem public protests against the regime. In June, Maduro’s government and the Guaidó opposition signed an agreement to coordinate COVID-19 relief efforts through PAHO. The deal, signed by Health Minister Carlos Alvarado, Dr. Julio Castro, who leads the National Assembly’s commission on the coronavirus, and PAHO will use government funds frozen by the U.S. and European governments to purchase personal protective equipment (PPE), medicines and medical equipment [34]. However, the situation is still very dire. As of August 10, 2020, PAHO has reported 241,811 cases and 4556 deaths in Venezuela compared to government reporting of 10,000 cases and 96 deaths [35]. Colombia closed its border with Venezuela on March 14, while Brazil did so partially on March 17 and allows only trucks carrying merchandise to cross in an effort to contain the spread of the virus. According to official data from June 15, 2020, the Brazilian government has sent 35,567 of 264,865 Venezuelans who requested migratory regularization (refuge or residence) to inland cities [36]. Given the intensity of this flow, the most serious consequence of COVID-19 for Venezuelans was the closure of Brazilian borders. Since the beginning of the pandemic, successive federal rules discriminate specifically against Venezuelan citizens and ban their entry into Brazilian territory even when they have a residence permit or a Brazilian spouse or children. The rules also establish the possibility of summary deportation, which is illegal under Brazilian law that guarantees migrants the right to a fair trial and the principle of due legal process [37]. The intention of the Brazilian government to take advantage of the health crisis to promote legislation that would limit migration is evident. The rise of the extreme right in 2018 to the presidency has brought a coarsening of policies and rhetoric toward human rights not seen since the end of the dictatorial military regime in 1985 [38].

In response to international and domestic movement restrictions put into place to respond to the COVID-19 pandemic as well as the lack of job opportunities in host countries, stigmatization, xenophobia, and violence, refugees have begun to return to Venezuela. Recent reports indicate that many refugees, including a recent influx of 60,000, are causing bottlenecks at border-control points leading to illegal crossings from Colombia. Venezuelans are met by security forces and mandatory quarantines lacking proper infection prevention control, PPE, and social distancing, creating a perfect environment for virus introduction and community transmission. Maduro has placed blame on his own people, noting these refugees returning home are responsible for recent surges in cases [39]. A senior member of Maduro’s party, Lisandro Cabello, was quoted saying “Any person who violates the immigration system and enters the country [illegally] will be considered a biological weapon [39].” Recent reports from outlets including the Human Rights Watch indicate that Venezuelan security forces are using the pandemic as cover to wage a “full force” campaign against dissenters, dozens of journalists, health professionals, human rights lawyers and government opponents have been arbitrarily detained and prosecuted since the COVID-19 state of emergency. As international organizations work to assist with the voluntary return, it is clear that the Maduro government’s priority is political survival and not the pandemic, creating an even stronger call for a coordinated response, accurate and timely data, and considerations for the welfare of Venezuelan migrants.

### Regional and global institution’s response

The geopolitical tensions surrounding Venezuelan political affairs only exacerbate the migrant crisis. While Latin American neighboring countries have taken on the responsibility of accepting Venezuelan migrants and refugees, the international community’s political strategies have deepened the core of the problem. The pandemic found regionalism at a time of decline. In the past, the Southern Common Market (MERCOSUR) was able to adopt rules on residency that made it possible to regularize the situation of tens of thousands of migrants in the region [40]. The Union of South American Nations (UNASUR) was considered a model in regional health diplomacy [41]. Currently, however, there is a total lack of coordination between the countries of the region resulting, among other factors, from the rise of conservative governments in countries such as Brazil, Colombia, Chile, and Ecuador not in favor of regional integration [42]. The political isolation of Venezuela also intensified as MERCOSUR suspended the country for violation of its democratic clause [43]. Countries that left UNASUR created the Forum for the Progress and Development of South America (PROSUR) [44]. These organizations have so far confined themselves to mere statements about the situation of Venezuelan migrants and have organized only modest initiatives relating to the pandemic in general [45]. PAHO is one of the few regional organizations involved as a partner in concrete initiatives related to the health of Venezuelan migrants and in Venezuela itself, which include distribution of tests, PPE, and training courses [46].

International economic sanctions placed on the Maduro regime to increase economic pressure and a decline in oil production are far outweighed by years of dismantling the private industrial sector and government corruption. Russia and China, which support the Maduro government, vetoed a resolution at the UN Security Council that called for free elections in February 2019. While the Lima Group, a 14-country bloc in the Americas, has repeatedly called for a peaceful transition of power, the Maduro government has been able to hold onto political and military control.

Efforts of cooperation to foster a coordinated response to the Venezuelan humanitarian process culminated in the “Quito Process” in September 2018, which formulated a road map and multilateral initiative of many Latin American countries that aims to help integrate Venezuelan migrants into host countries through coordinating domestic policies. The Quito Process has so far resulted in two declarations and one action plan in which governments in the region have sought to meet the most critical needs of Venezuelan migrant populations in the different countries. In addition, the UNHCR and International Organization for Migration (IOM) launched the Regional Refugee and Migrant Response Plan in December 2018, which is a first of its kind operational and coordination strategy and an appeal for $738 million in funding to support over two million Venezuelans in the region and half a million people in host communities.

With Venezuela’s political system remaining in deadlock, her people continue to suffer. The international divide hampers decision-making at multilateral organizations such as the International Monetary Fund and the World Bank, where divided recognition of the Venezuelan government blocks the country from receiving desperately needed loans. The international tensions politicize aid to the Venezuelan people who are in dire need of humanitarian aid due to food shortages and lack of adequate healthcare access. The lack of cooperation tempers data collection and information gathering on the ground. Disparate data only paint an incomplete picture and could miss crucial needs of Venezuelans both inside and outside of Venezuela. As the crisis continues, Venezuelans continue to migrate to neighboring Latin American countries, carrying with them the ramifications of their broken health system. Migration is a social determinant of health primarily because the conditions of the migratory path determine the health of migrants and, as discussed, the exportation of infectious diseases from Venezuelans across international borders has had serious repercussions on the health systems of the neighboring countries. The severity of the COVID-19 pandemic globally further illustrates the need for data collection and information sharing.

## Conclusion

In his seven years in power, Mr. Maduro has overseen the collapse of Venezuela’s health care system, the destruction of the national economy, and a marked increase in the country’s international isolation. Venezuela now ranks second, only to Syria, for countries with forcibly displaced populations. The difficulty in handling the situation does not mean that countries should close their borders to migrant populations. Rather, it is imperative that cross-border political cooperation organizes for a joint investigation and response to migrant health needs. Coordinated access to migrants could provide information to deepen international response. These mechanisms and health structures can be a combination of official and unofficial; to provide participation of public institutions for the stability of these structures for long-term solutions and to activate community-based institutions to better understand the perceived needs of the people. Furthermore, health services must adapt to become more inclusive of migrant’s health needs, as well-established structures that can overcome the financial barriers to health care access. As the COVID-19 pandemic continues to spread, the existing paradigms on how to deal with crises in and around Venezuela continue to change. With cooperation and joint efforts between countries, along with support rather than conflict from the international community, migrant health and border health challenges resulting from the Venezuelan migrant crisis can be better controlled.

## Data Availability

Data on the number of refugees and migrants from Venezuela are available from the Response for Venezuelans Coordinating Platform at https://r4v.info/. Data on the number of confirmed COVID-19 cases are available from WHO’s Coronavirus Disease (COVID-19) Dashboard Available from: https://covid19.who.int

## Abbreviations

IMF: International Monetary Fund
UNHCR: United Nations High Commission for Human Rights
WHO: World Health Organization
PAHO: Pan-American Health Organization
COVID-19: Novel coronavirus
PPE: Personal protective equipment
MERCOSUR: Southern Common Market
PROSUR: Progress and Development of South America
IOM: International Organization for Migration

## References

[1] Nelson RM (2018). Venezuela’s Economic Crisis: Issues for Congress [Internet].

[2] Office of the United Nations High Commissioner for Human Rights (2018). Human rights violations in the Bolivarian Republic of Venezuela: a downward spiral with no end in sight [internet].

[3] Seelke CR (2020). Venezuela: Political Crisis and U.S. Policy [Internet].

[4] Bello UCA. Encuesta Nacional de Condiciones de Vida (ENCOVI) 2019–2020 [Internet]. ENCOVI. 2020; Available from: https://www.proyectoencovi.com/informe-interactivo-2019.

[5] The Lancet (2018). The collapse of the Venezuelan health system. Lancet.

[6] Ministerio del Poder Popular del Despacho de la Presidencia y Seguimiento de la Gestión de Gobierno, Venezuela (2020). Arsenal terapéutico incluye suministro de cloroquina para pacientes contagiados, sospechosos y personal de salud [Internet].

[7] Paniz-Mondolfi AE, Sordillo EM, Márquez-Colmenarez MC, Delgado-Noguera LA, Rodriguez-Morales AJ (2020). The arrival of SARS-CoV-2 in Venezuela. Lancet.

[8] Médicos por la Salud. Encuesta Nacional de Hospitales [Internet]. [cited 2020 Aug 10]. Available from: https://www.encuestanacionaldehospitales.com.

[9] Daniels JP (2020). Venezuelan migrants “struggling to survive” amid COVID-19. Lancet.

[10] Escalona J. FMV: Más de 30.000 médicos se han ido de Venezuela #12Sep. El Impulso.com [Internet]. 2019 Sep 12 [cited 2020 Aug 10]; Available from: https://www.elimpulso.com/2019/09/12/fmv-30-000-medicos-se-han-ido-de-venezuela-12sep/.

[11] Sarmiento H, Cobo OB, Morice A, Zapata R, Benitez MV, Castillo-Solórzano C (2011). Measles outbreak in Venezuela: a new challenge to Postelimination surveillance and control?. J Infect Dis.

[12] Paniz-Mondolfi AE, Tami A, Grillet ME, Márquez M, Hernández-Villena J, Escalona-Rodríguez MA (2019). Resurgence of vaccine-preventable diseases in Venezuela as a regional public health threat in the Americas. Emerg Infect Dis.

[13] UNAIDS. Venezuela: Overview [Internet]. UNAIDS. 2020; [cited 2020 Feb 27]. Available from: https://www.unaids.org/en/regionscountries/countries/venezuela.

[14] Daniels JP (2017). Venezuela’s economic crisis hampers HIV/AIDS treatment. Lancet.

[15] Page KR, Doocy S, Reyna Ganteaume F, Castro JS, Spiegel P, Beyrer C (2019). Venezuela’s public health crisis: a regional emergency. Lancet.

[16] Selee A, Bolter J (2020). An uneven welcome: Latin American and Caribbean responses to [internet].

[17] Grillet ME, Hernández-Villena JV, Llewellyn MS, Paniz-Mondolfi AE, Tami A, Vincenti-Gonzalez MF (2019). Venezuela’s humanitarian crisis, resurgence of vector-borne diseases, and implications for spillover in the region. Lancet Infect Dis.

[18] Doocy S, Page K, Broner TT (2019). Venezuela’s humanitarian emergency | large-scale UN response needed to address health and food crises [internet].

[19] Pan American Health Organization/World Health Organization (2018). Epidemiological Update: Malaria. 30 January 2018.

[20] Tuite AR, Thomas-Bachli A, Acosta H, Bhatia D, Huber C, Petrasek K (2018). Infectious disease implications of large-scale migration of Venezuelan nationals. J Travel Med.

[21] Pan American Health Organization/World Health Organization (2018). Epidemiological Update: Measles. 20 August 2018.

[22] Conselho Nacional dos Direitos Humanos (2018). Relatório das violações de direitos contra imigrantes venezuelanos no Brasil, January 2018 [Internet].

[23] Rodrigues I d A, Cavalcante JR, Faerstein E (2020). Pandemia de Covid-19 e a saúde dos refugiados no Brasil. Physis.

[24] de Arruda-Barbosa L, Sales AFG, de Souza ILL (2020). Reflexos da imigração venezuelana na assistência em saúde no maior hospital de Roraima: análise qualitativa. Saude Soc.

[25] Pérez Ortega R (2020). ‘We’re losing an entire generation of scientists.’ COVID-19’s economic toll hits Latin America hard. Science [Internet].

[26] Freitez A, Mazuera R, Delgado M, Nava B (2020). Situación de migrantes venezoelanos recientes en el contexto del Covid-19. Reporte Situacional 2020 [Internet].

[27] Nunes J, Ventura D, Lotta GS. Brazil: Jair Bolsonaro’s strategy of chaos hinders coronavirus response. Conversation [Internet]. 2020; Available from: https://theconversation.com/brazil-jair-bolsonaros-strategy-of-chaos-hinders-coronavirus-response-136590.

[28] WHO. Brazil situation [Internet]. Coronavirus Disease (COVID-19) Dashboard. [cited 2020 Aug 16]. Available from: https://covid19.who.int/region/amro/country/br.

[29] de Estado da Saúde S (2020). Boletim Epidemiológico.

[30] Conectas Direitos Humanos (2020). How migrants and refugees in Brazil are surviving in the pandemic [Internet].

[31] Jarochinski Silva JC, Jubilut LL (2020). Venezuelanos no Brasil e a Covid-19. Migrações internacionais e a pandemia de Covid-19.

[32] Vilela PR. Hospital de campanha inicia atendimento de refugiados em Roraima: Agência Brasil [Internet], Brasilia; 2020. [cited 2020 Aug 26]; Available from: https://agenciabrasil.ebc.com.br/saude/noticia/2020-06/hospital-de-campanha-inicia-atendimento-de-refugiados-em-roraima.

[33] Transparencia Venezuela. Maduro no escucha a la CIDH y aprovecha el COVID-19 para censurar [Internet]: Transparencia Venezuela; 2020. [cited 2020 Aug 10]. Available from: https://transparencia.org.ve/maduro-no-escucha-a-la-cidh-y-aprovecha-el-covid-19-para-censurar/.

[34] Venezuela’s Maduro joins hands with Guaido in fight against virus (2020). Al Jazeera [Internet].

[35] WHO. WHO Coronavirus Disease (COVID-19) Dashboard [Internet]. [cited 2020 Aug 10]. Available from: https://covid19.who.int.

[36] Government of Brazil. A Operação Acolhida [Internet]. Operação Acolhida. [cited 2020 Aug 13]. Available from: https://www.gov.br/acolhida/historico/.

[37] Conectas Direitos Humanos (2020). Covid-19 propels number of rules issued by the federal government [Internet].

[38] Ventura D (2020). Between science and populism: Brazil and COVID-19. Chatham House [Internet].

[39] Herrero AV, Faiola A, Zuñiga M. As coronavirus explodes in Venezuela, Maduro’s government blames ‘biological weapon’: the country’s returning refugees: Washington Post [Internet]; 2020. Available from: https://www.washingtonpost.com/world/the_americas/coronavirus-venezuela-migrant-maduro/2020/07/19/582c659c-c518-11ea-a99f-3bbdffb1af38_story.html.

[40] Braz AM (2018). Migration governance in South America: the bottom-up diffusion of the residence agreement of Mercosur. Rev Adm Pública Fundação Getulio Vargas.

[41] Riggirozzi P (2015). Regionalism, activism, and rights: new opportunities for health diplomacy in South America. Rev Int Stud.

[42] Riggirozzi P. Coronavirus y el desafío para la gobernanza regional en América Latina. AC [Internet]. 2020; [cited 2020 Aug 26]; Available from: https://www.fundacioncarolina.es/ac-12-2020/.

[43] Dieguez J. Suspensión de Venezuela en el MERCOSUR [Internet]. MERCOSUR. 2017; [cited 2020 Aug 26]. Available from: https://www.mercosur.int/suspension-de-venezuela-en-el-mercosur/.

[44] Sobre PROSUR [Internet]. PROSUR. [cited 2020 Aug 26]. Available from: https://foroprosur.org/sobre-prosur/.

[45] Malacalza B, Hirst M (2020). ¿Podrá reinventarse el multilateralismo? El orden internacional y el coronavirus | Nueva Sociedad [Internet]. Nueva Sociedad | Democracia y política en América Latina.

[46] Pan American Health Organization/World Health Organization (2020). Pan American health organization response to COVID-19 in the Americas [internet].

